# Autologous conditioned serum applications in the treatment of musculoskeletal diseases: a narrative review

**DOI:** 10.2144/fsoa-2021-0088

**Published:** 2022-01-12

**Authors:** Seyed Ahmad Raeissadat, Seyed Mansoor Rayegani, Nafisseh Jafarian, Mina Heidari

**Affiliations:** 1 Physical Medicine & Rehabilitation Department, Clinical Research Development Center of Shahid Modarres Educational Hospital, School of Medicine, Shahid Beheshti University of Medical Sciences, District 2, Sa'adatabad Boulevard, 19987 34383, Tehran, Iran; 2 Physical Medicine & Rehabilitation Department & Research Center, School of Medicine, Shahid Beheshti University of Medical Sciences, District 1, Daneshjou Boulevard, 19839 69411, Tehran, Iran

**Keywords:** autologous conditioned serum, Interleukin-1 receptor antagonist protein, literature review, musculoskeletal diseases, orthokine, osteoarthritis

## Abstract

**Aim:**

Autologous conditioned serum has been studied as a treatment option in musculoskeletal disorders and resulted in varying outcomes. This study aims to pool the current data on this matter.

**Materials & methods:**

Major databases were searched for the topics, and after screening the results, the final 21 papers (level of evidence I or II) were included.

**Results & conclusion:**

This study showed a major focus of the literature on the effectiveness of autologous conditioned serum in osteoarthritis, in which there is much high-quality evidence suggesting its safety and efficacy. Also, some of the available experiments are assessing its application in tendinopathies and radiculopathies which, despite positive results, recommend further evaluations on this topic.

## Lay abstract

Autologous conditioned serum (ACS) is a cell-free blood product rich in anti-inflammatory components; this study aims to provide a review of the available data on its clinical applications in musculoskeletal disorders. The most studied subject was joint diseases and mainly osteoarthritis, for which some high-quality papers suggested ACS to be an effective and safe option. A few papers assessed the effectiveness of ACS in soft tissue injuries like tendinopathies and plantar fasciitis, and also cervical and lumbar radiculopathies in human subjects; despite some positive results, a further high-quality investigation is required on this matter.

Musculoskeletal disorders (MSKDs) are the second most common cause of disability worldwide, measured by years lived with disability; and from 1990 to 2010 an increase of 45% have been estimated in the disability due to MSK disorders, in particular, osteoarthritis (OA), and is expected to continue to rise with an increasingly obese, sedentary and aging population [1,2]. Regarding the significant prevalence and wide variety of this category of illnesses (from tendon injuries in young athletes to degenerative disorders in the elderly), and also considering the chronic and mostly non-curable nature of some, finding an effective treatment has always been of great importance.

Although there are different therapeutic modalities used to control the symptoms of MSKDs, most of them cannot halt the progression of the disease. Surgical interventions put aside, pharmacological treatments (such as NSAIDs, SNRIs, opioids, etc.) and nonpharmacological modalities (exercise therapy, aqua therapy, physical therapy, etc.) can improve function in most patients. Regenerative medicine and autologous blood product injections are also some of the least invasive methods with acceptable therapeutic results in many MSKDs [3–13]. Regarding the contribution of biologic alongside biomechanical factors, to orthopedic disease pathogenesis, the possible effects of cytokine inhibitors and growth factors were proposed in the 1970 and 80s. IL-1 appears to be an important one, among the cytokines identified in MSKDs. IL-1 receptor antagonist (IL-1ra) is a competitive receptor antagonist of IL-1, and it is hypothesized that the local IL-1ra concentration is too low in degenerative diseases to inhibit the destruction of cartilage, muscles, spine tissue and other joint structures [14–16].

In the search for disease-modifying treatment options rather than already-known symptom-modifying ones, autologous conditioned serum (ACS) was introduced in the 1990s; with the venous whole blood incubated in the presence of specialized glass spheres to initiate monocyte activation. The resulting conditioned serum contains elevated levels of various anti-inflammatory cytokines, such as IL-1ra, IL-10 and TGF-B1 (up to 7.9, 3 and 14.9 folds, respectively, in comparison to unconditioned serum) [15–18].

Despite the considerable attention gained by this new method, specifically in knee OA treatment, it is noteworthy that ACS application is not yet approved by US FDA (trial available at: https://clinicaltrials.gov/ct2/show/NCT03850080).

Through the last two decades, the effectiveness and safety of ACS for MSKDs have been investigated in many studies with animal and human subjects, as well as *in vitro* and *in vivo* [9,16,19–22]. The present study aims to provide a collective review of the current information available on this topic, focusing on human-based high-quality research.

## Materials & methods

A comprehensive literature review was conducted separately by two researchers: physical medicine and rehabilitation residents N Jafarian and M Heidari – on the major electronic databases, PubMed (NLM), Cochrane Library (CENTRAL), Science Direct, Scopus, Web of Science, Embase and LILAC, as well as Google Scholar search engine. An expert librarian assisted in performing a manual search in nonelectronic bibliographic sources.

The search strategy contained different combinations of the following keywords: “autologous conditioned serum”, “ACS”, “Interleukin-1 receptor antagonist protein”, “IRAP”, “Orthokine”, “musculoskeletal diseases”, “sports injuries”, “joint diseases”, “osteoarthritis”, “tendinopathy”, “ligament injuries”, “soft tissue injuries”, “radiculopathy”, “discopathy pain” and “treatment”.

The search queries included combined word text with truncations, wildcard operators (“*”), proximity and Boolean operators (“AND”, “OR”, “NOT”, “NEAR” and “ADJ”), as well as the specific controlled vocabulary for each database if available (e.g., MeSH terms in PubMed).

The information sources were searched for published and unpublished documents in the timeframe from January 2000 up to April 2021, with no limits applied for language or country to maximize the sensitivity.

Exclusion criteria were considered as the application of concomitant therapeutic methods alongside ACS, such as surgical interventions, physical modalities or pharmacotherapy; studies with animal models or *in vitro* subjects, and those in languages other than English or Persian.

After removing duplicates, the articles were screened by title and then abstract using a reference manager application (EndNoteX9); any disagreements were resolved by discussion or were referred to the senior physicians (SA Raeissadat and SM Rayegani). The most relevant studies were classified according to the subject and date of the study, and for better results, only those with a level of evidence of I or II (explained below) were finally included and discussed.

The final eligible studies were categorized based on the diseases, and the quality assessment and further discussion were performed in chronological order and regarding the level of evidence according to the criteria of the American Association of Physical Medicine & Rehabilitation, an adaptation of those proposed by the *Journal of Bone and Joint Surgery* [23]:

- Level I – randomized controlled trials or systemic review of level I randomized controlled trials
- Level II – prospective cohort studies, poor-quality randomized controlled trials, systemic reviews of level II studies, or nonhomogenous level I studies
- Level III – case-control studies, retrospective cohort studies, systemic reviews of level III studies
- Level IV – case series
- Level V – expert opinion

In addition, the GRADE quality assessment system has also been applied and integrated into the process wherever possible. This tool categorizes the studies in four categories: high, moderate, low, and very low; and provides either “strong” or “weak” recommendations [24,25].

## Results & discussion

**Figure 1.**
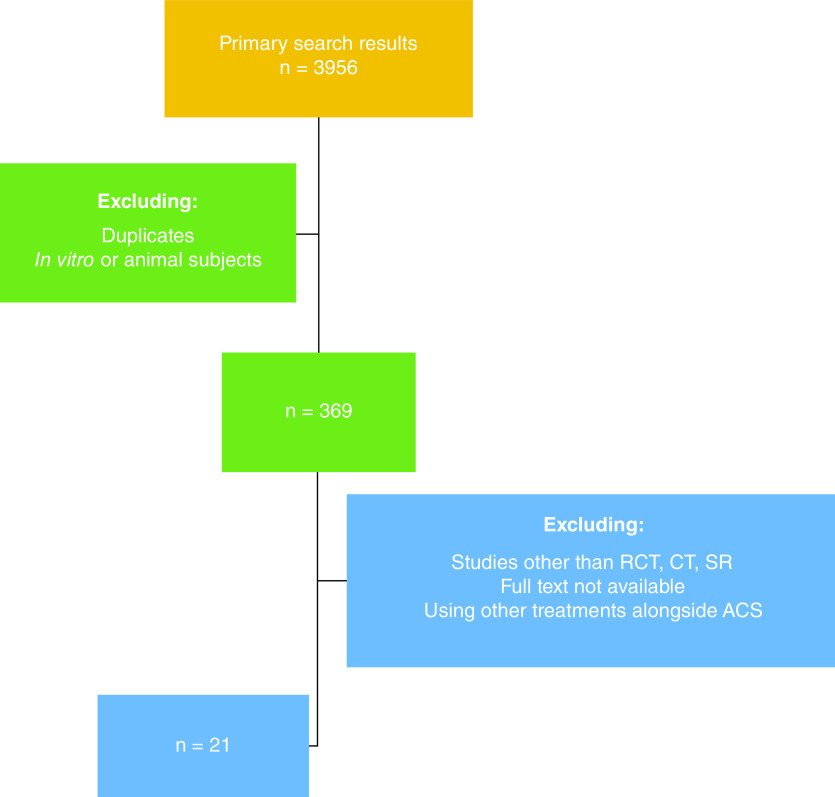
Study selection flow chart.

The primary search for applications of ACS for the treatment of musculoskeletal diseases in the period of January 2000 to April 2021 resulted in 3956 papers, after deduplication and excluding animal or *in vitro* experiments, 369 remaining studies were screened for inclusion criteria. Considering the inclusion/exclusion criteria and level of evidence, the final number of 21 studies investigating the applications of ACS for MSKDs with the level of evidence I or II, performed on human subjects – *in vivo* – with no concomitant treatments applied, and were enrolled (Figure 1).

**Figure 2.**
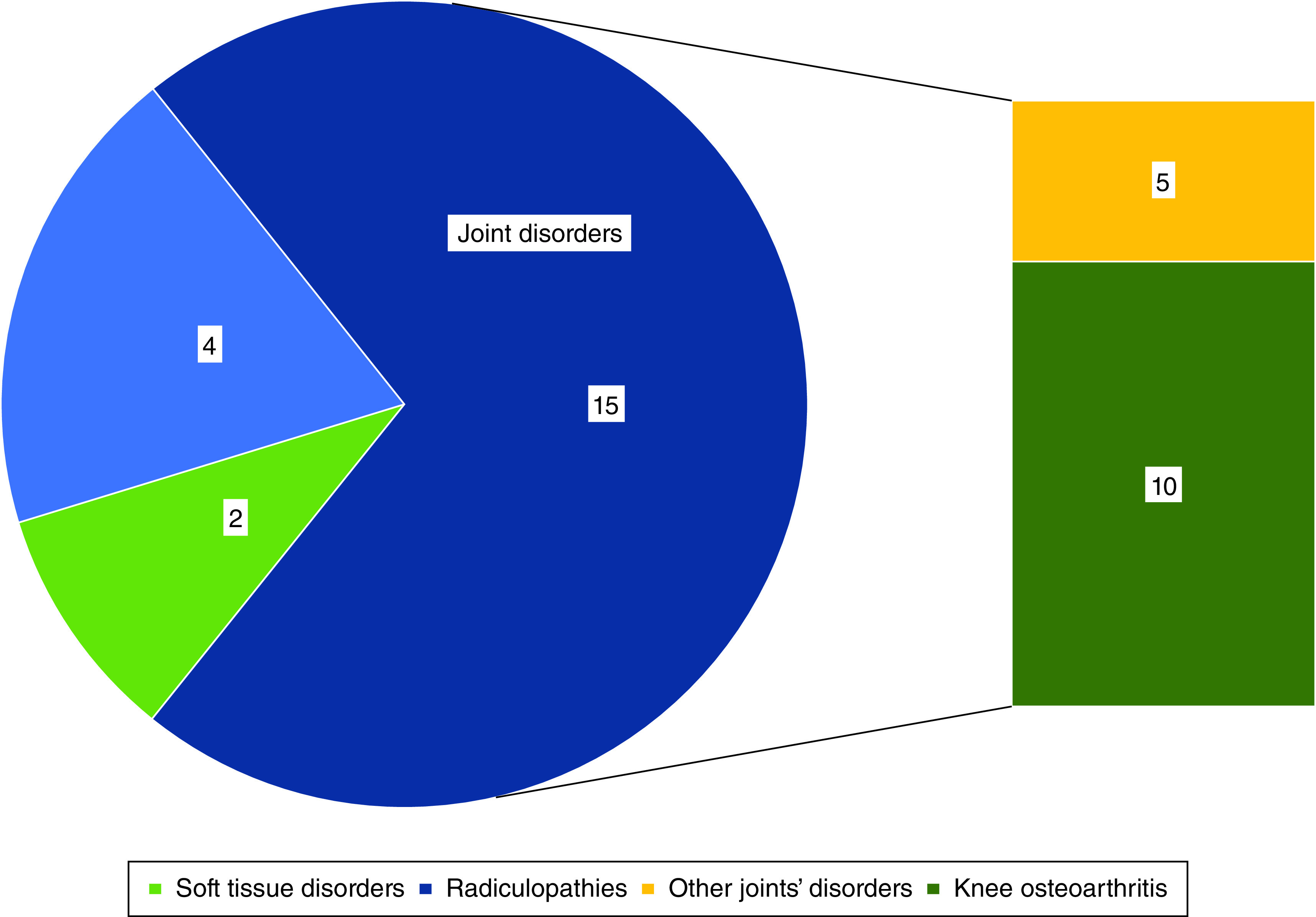
Number of final studies based on the focused topic.

Of the 21 included articles, 15 explored ACS effectiveness in joint-related diseases (mostly osteoarthritis), two of them probed the applications of this method in soft tissue disorders (tendinopathies and achillodynia) and four investigated its possible role in the treatment of other MSKDs, mainly radiculopathies (Figure 2).

Regarding the wide spectrum of musculoskeletal diseases and different applications of ACS, each will be discussed in a separate section, according to the level of evidence and in chronological order.

## Joint disorders

Joint disorders and especially OA are the most common and most studied applications of ACS.

Ten pieces of research focused on knee OA, with the level of I or II, nine of which suggested ACS as an effective treatment for this disease. Four of these studies were designed as randomized control trials (RCTs) with high quality according to the GRADE tool, five as clinical trials, and one as a systematic review, considered as moderate to low-quality evidence. (summarized in Table 1 & 2).

**Table 1.** Summaries of randomized controlled clinical studies on the effectiveness of autologous conditioned serum for knee osteoarthritis treatment.

Study (year)	Study design/level of evidence	Sample size/disorder	Intervention/ comparison	Outcome measures	Follow up	Results
Yang (2008)	RCT-DB/I	167/KOA	ACS/saline	WOMAC, KOOS	3, 6, 9, 12 months	Comparable WOMAC but ACS significantly superior in KOOS. Currently, it cannot yet be recommended for the treatment of OA
Baltzer (2009)	RCT-DB/I	376/KOA	ACS/HA-saline	WOMAC, VAS, SF-8	7, 13, 26 weeks + 2 years	ACS significantly superior in all outcome measures and all time points
Hashemi (2019)	RCT-DB/I	60/KOA	ACS/HA	KOOS, WOMAC	6 months	The superiority of ACS in KOOS and WOMAC
Pishgahi (2020)	RCT/I	92/KOA	ACS/PRP, Dextrose	WOMAC, VAS	0, 1, 6 months	Improved VAS and WOMAC in both ACS and PRP groups, more significantly in ACS group

ACS: Autologous conditioned serum; KOOS: Knee injury and osteoarthritis Outcome Score; PRP: Platelet rich plasma; RCT: Randomized control trial; VAS: Visual analog scale; WOMAC: Western Ontario and McMaster Universities Arthritis Index.

**Table 2.** Summaries of nonrandomized control trial studies on the effectiveness of autologous conditioned serum for knee osteoarthritis treatment.

Study (year)	Study design/level of evidence	Sample size/disorder	Intervention/ comparison	Outcome measures	Follow up	Results
Ajrawat (2019)	SR of 3 RCTs and 5 CTs/II	592/KOA	ACS	WOMAC, KOOS, VAS, SF-36	-	ACS can improve pain and functional outcome in patients with mild to moderate knee OA
Fathalla (2014)	CT/II	30/KOA	ACS	WOMAC	1, 3, 4 weeks	Highly significant improvement in all scores
Rutgers (2015)	CT/II	20/KOA (previous placebo group of another RCT)	ACS	VAS, KOOS, KSCRS, WOMAC	3, 6, 9, 12 months	Initial improvement in pain (VAS) score but after 12 months the clinical results were back to those after placebo treatment
Shirokova (2017)	CT/II	123/KOA	ACS/PRP	WOMAC, VAS	1, 3 months	ACS had a longer-lasting effect and was more effective in pain reduction and function improvement in patients with apparent clinical synovitis
Kilinc (2019)	CT/II	33/bilateral symmetrical KOA	ACS	VAS, KSS, KOOS	12 months	Significant clinical improvement in VAS, KOOS and KSS scores
Vitali (2020)	CT/II	15/KOA	ACS	VAS, WOMAC, KSS	1, 2, 3 weeks 1, 6 month	Significant improvement in both clinical and functional scales

ACS: Autologous conditioned serum; CT: Clinical trial; DB: Double blind; HA: Hyaluronic acid; KOA: Knee osteoarthritis; KOOS: Knee injury and osteoarthritis outcome score; KSS (=KSCRS): Knee Society Clinical Rating System; OA: Osteoarthritis; PRP: Platelet-rich plasma; RCT: Randomized control trial; SF: Short form; VAS: Visual analog scale; WOMAC: Western Ontario and McMaster Universities Arthritis Index.

Baltzer *et al.* conducted a well-designed randomized double-blind placebo-controlled clinical trial on 376 patients diagnosed with knee OA (KL II–III). They compared the effectiveness of intra-articular (IA) ACS, HA and saline (placebo) assessed by Western Ontario and McMaster Universities Arthritis Index (WOMAC), visual analog scale (VAS), short form (SF)-8 as outcome measures; after 7, 13 and 26 weeks follow up, they concluded that the effects of ACS were significantly superior to hyaluronic acid and saline for all outcome measures and all of the time points. As their findings revealed VAS changes in the ACS group as 69.6 (±13.10) decreased to 29.5 (±22.58 by week 26) and in the hyaluronic acid group as 68.3 (±12.81) to 49.3 (±25.9) in the same time point. The frequency of adverse events was comparable in the ACS and saline groups.

Furthermore, the researchers performed a 2-year observer-blinded- follow-up visit, which showed persistent therapeutic effects of ACS. Although, this study could not distinguish these effects to be either symptom or structure modifying [18].

In Iran, Pishgahi *et al.* also conducted an RCT on 92 patients with knee osteoarthritis, allocated in three demographically homogenous groups, to compare the effectiveness of IA-ACS with platelet rich plasma (PRP) and dextrose prolotherapy. This study resulted in significant improvement in ACS and PRP groups (ACS more than PRP) in both outcome measures for pain and function (VAS and WOMAC) in 1- and 6-month follow up; with no major adverse effects. The authors stated that ACS can be more effective than PRP in pain reduction and function improvement, and regarding its less variable processing and less reported side effects, it is a better choice for knee OA patients [26].

The other paper on this subject was the double-blinded RCT by Hashemi *et al.*, in which, 60 patients with knee OA were randomly divided into two homogenous groups of 30, to compare the effects of IA-ACS with hyaluronic acid. This study revealed the superiority of ACS in knee injury and osteoarthritis outcome score (KOOS) and WOMAC scores in 6-month follow up. They reported KOOS (signs) changes as 41.9 ± 2.5 to 71.7 ± 14.3, and the KOOS (daily activities) as 37.9 ± 8.1 to 72.6 ± 9.2 in 6 months [27].

Furthermore, Ajrawat *et al.* studied the effectiveness of IA autologous IL-1 antagonist blood products for knee OA in a systematic review; they included three high-quality RCTs and five clinical trials on the subject (level of evidence II to IV) with a total number of 592 patients. It should be noted that a number of the papers included in his study have already been discussed in the present review. Regarding outcome measures of WOMAC, KOOS, VAS and SF-36 this study disclosed that this therapeutic method can improve pain and functional outcomes in patients with mild to moderate knee OA [28].

The other clinical trial by Shirokova *et al.* compared IA-ACS with PRP in 123 female patients diagnosed with knee OA. Patients were allocated in each group regarding age, BMI and presence of synovitis, for baseline comparability. This study showed, despite the positive effect of both interventions, that ACS had a longer-lasting effect (to the 3rd month follow up), and also it was more effective in pain reduction and function improvement (VAS and WOMAC scores) in patients with apparent clinical synovitis; VAS changes in ACS group (+ moderate synovitis) were 64 ± 12.8 to 34 ± 17.4 and in PRP group 59.4 ± 17.4 to 54.7 ± 16.4 in 3 months. So the preferred method of treatment in regards to anti-inflammatory and lasting effect was suggested to be ACS rather than PRP. The notable point in this study was that the authors combined biochemical evaluations with clinical outcome measures. Joint aspirations were performed at baseline and one month after the treatment, assessing the synovial fluid viscosity, the content of IL-1a and IL-b, and nitrate concentration; all calculated to be significantly different in the ACS group. Viscosity was increased with a steady slope in the ACS group, and it showed an increase and then decreased until day 90 in the PRP group. IL-1a and IL-b were respectively higher and lower in the ACS group, and the N03 level was significantly dropped at all-time points in the ACS group, but showed a decline in one month and then increased in 3 months follow up. This study provided data for the superiority of ACS in both clinical and biochemical outcomes [29].

Another clinical trial by Kilinc evaluated the effectiveness of inferior alveolar (IA) ACS in 33 patients with bilateral (and symmetrically progressed) knee OA. After a 1-year monitoring period, they concluded that IA ACS had significant clinical improvement in VAS, KOOS and Knee Society Clinical Rating System scores; and can be an alternative treatment in knee OA patients [30].

Vitali *et al.* also explored pre- and post-treatment scales of VAS, WOMAC and Knee Society Clinical Rating System in 15 patients with knee OA, after 4 IA injections of ACS. Their study as others mentioned above revealed significant improvement in both clinical and functional scales after the intervention. Despite few adverse effects (pain and swelling and one case of joint rigidity after injection), the authors suggested ACS as a safe and tolerable as well as effective therapeutic intervention for knee OA [31]. In another research conducted in Egypt, Fathalla *et al.* investigated the effectiveness of IA ACS in 30 patients with knee OA (KL grade I–III) and reported significant improvement in all WOMAC scores, which persisted until the third month of follow up [32].

Also, Rutgers recruited the placebo group of an earlier RCT on the effectiveness of IA ACS for knee OA treatment; 20 of those 74 patients opted for further treatment with ACS and they were followed up for 12 months. This study revealed an initial improvement in pain (VAS) score but after 12 months the clinical results were back to those after placebo treatment. Although due to the low power of this study the results should be cautiously interpreted [33].

On the other hand, some researches did not result in similar to those mentioned above. Yang *et al.* evaluated this method in the treatment of symptomatic knee OA in a high-quality randomized, multicenter, double-blind, placebo-controlled trial. They treated 167 patients with six doses of IA injections of either ACS or saline; VAS, WOMAC and KOOS indexes were assessed in 3, 6, 9 and 12 months post-treatment. The results showed consistently higher levels of improvement in all outcome measures in the ACS group, although it was statistically significant only in KOOS and KOOS sport; the authors thus concluded that despite the effectiveness of this method, currently it cannot yet be recommended for the treatment of OA [34].

Regarding the overall adverse events profile, three of these studies (Kilinc, Pishgahi and Shirokova) observed no adverse events in the subjects; Vitali *et al.* reported five patients with local pain, three with joint swelling and one with rigidity. The study by Baltzer *et al.* also mentioned that two out of 107 subjects in the Saline group, five out of 135 in the hyaluronic acid group. In the ACS group, only mild to moderate local symptoms occurred and were resolved in a few hours. The frequency of adverse events was not reported in Hashemi and Fathalla’s studies.

This overview suggests a low-risk profile for adverse events in ACS therapy for knee OA.

It should be noted that ‘efficacy’ in knee OA treatment is considered in terms of optimizing the most commonly used outcome measures such as VAS for pain and WOMAC for function assessment. Although an overall consensus on the minimal clinically important difference for these variables is still not reached, some studies are reporting the most reliable measures. As the current standard published in the literature, the minimal clinically important difference for VAS (out of 10 scales) is reported 1.37 [35] and WOMAC (function) is measured as 5.89–8.1, and for KOOS there is no specific measure reported yet [36].

Additionally, in the clinical trial by Baltzer *et al.*, the therapeutic effects of ACS versus ACS + cortisone versus ACS + cortisone + recombinant IL-1 receptor antagonist protein (IL-1ra) were compared in a total number of 119 patients with hip OA. After 14 months of follow up, they disclosed that although there was a significant improvement in VAS in all three groups (regardless of the disease severity), neither cortisone nor cortisone + IL-1ra did not increase the beneficial effects of ACS in these patients; and of course, further investigations with high-quality RCTs are suggested on this topic [17].

Coxarthrosis (CA) is another joint disorder that has been subjected to this intervention. Noskov conducted a clinical trial on 54 patients with CA and monitored them for 12 months after IA injection of ACS versus HA, evaluating pain and functional improvement (VAS and WOMAC). They reported significant superiority in the outcome measures after 3 months in the ACS group and suggested this method to be a proper alternative to HA in CA with longer-lasting results [37].

Also, a clinical trial comparing IA ACS versus low molecule HA for CA was conducted by Shirokova in 2015. This study included 60 patients allocated in two comparable groups in terms of demographic data and the clinical status of the disease. At months of follow up, the ACS group showed more improvement in pain (VAS) and WOMAC functional scale, although the overall clinical efficacy by Lequesne index at 6 months’ time point was comparable in both groups [38].

Glenohumeral OA response to IA ACS was also investigated in a clinical trial by Aartsen *et al.* They recruited 40 shoulders in 36 patients with painful GH-OA, the radiological type determined according to Walch classification (centered/decentred). Primary outcome measures were passive, and active range of shoulder motion, total Shoulder Pain and Disability Index (SPADI), the American Shoulder and Elbow Surgeons Standardized Shoulder Assessment Form (ASES), and the constant score. Post-intervention measurements were performed 3–12 months after treatment. The results showed significant improvement in both pain and disability after treatment with ACS, and it was also mentioned that the therapeutic effects were not related to the radiological classification of type or degree of the disease. The authors declared that this intervention can postpone the need for shoulder replacement surgery [8].

Of other joint disorders, the meniscal injury was assessed in a clinical trial on 47 patients (mean age = 48.6 years) with heterogeneous knee meniscus lesions (76.6% traumatic knee injury). In this study, Strumper *et al.* evaluated Oxford Knee Score and structural changes with the MRI Boston Leeds Osteoarthritis Knee Score at baseline and 6 months after IA injection of ACS. The results showed a significant improvement in both clinical and structural outcome measures in 6 months follow up; the authors suggested this intervention as an effective method for treatment of knee pain associated with meniscal injuries; although due to the small sample size, further investigations are recommended [39].

## Soft tissue disorders

Soft tissue pathologies are another prevalent and disabling entity of MSKDs, and ACS has been gaining attention in the treatment of these conditions. Although it has shown efficacy in the treatment of tendon and muscle injuries in animals, high-quality studies on human *in vivo* models are still lacking.

Damjanov *et al.*, in a 24-week randomized double-blind study, compared the efficacy and safety of ACS with glucocorticoid (betamethasone) injections in 32 patients with chronic supraspinatus tendinopathy. Outcome measures of pain intensity (VAS) and Constant Shoulder Score (CSS) assessed at weeks 0, 4 and 24; showed pain improvement in 4 weeks and significant improvement after 24 weeks of monitoring in the ACS group compared with the glucocorticoid group. CSS was also improved in both groups but at the 24-week time point, ACS patients reported significantly greater CSS improvements than glucocorticoid patients. Also, adverse events (n = 8) were reported in betamethasone patients. This study suggested ACS as a safe and effective treatment option for chronic rotator cuff tendinopathy [40].

Another clinical trial (by Majewski *et al.*) evaluated the efficacy of ACS local injection on 25 patients (mean age 50 years old) with MRI-confirmed achillodynia (i.e., tendinous effusion, thickening); symptoms persisting after 6 months of physical therapy. After three weekly injections, patients were instructed to avoid using NSAIDs for 6 months and sports for 4 weeks, full weight-bearing was permitted immediately and eccentric exercises after 4 weeks of injection. Outcome measures of The Victorian Institute of Sports Assessment-Achilles questionnaire (VISA-A) and a follow-up MRI was performed at 0 and then 6 months, which showed 100% structural improvement alongside 100% pain reduction and 88% regaining sports activity. Despite positive outcomes, regarding the study design and small sample size, as it is considered a piece of relatively low-quality evidence according to the GRADE system, its results should be interpreted cautiously [41].

## Radiculopathies

Of other applications of ACS, radiculopathies are mostly explored; although these were mostly pilot studies with small sample sizes and require further investigations via well-designed researches including a larger number of subjects.

In a pilot study by Godek *et al.* on 15 patients with MRI-confirmed single-level lumbar disc herniation with clinical signs of radiculopathy, six doses of ACS were injected (ultrasound-guided posterolateral approach to the intervertebral foramen). Patients were assessed in 1 and 3 months after the last injection for pain intensity with a scoring system and a VAS scale, and for radicular edema with the following clinical tests: one-leg stance (OLS test) and straight leg raise (SLR test); the disability level was measured with the Oswestry Disability Index (ODI). This study showed significant improvement in pain and disability in 13 patients, two of them underwent surgery due to increased pain, and no radicular damage was reported. The authors concluded ACS can be a potential treatment for radicular compression, although many further stronger studies are required on this subject [12].

In addition, Becker conducted a prospective, double-blind, reference-controlled trial on 84 patients; 32 of which underwent epidural perineural injections with ACS, and two groups of 27 and 25 were treated with 5 and10 mg triamcinolone, respectively. After 3 weekly injections, patients were followed up to 6 months and evaluated for VAS of lower back pain and the ODI. All patients showed remarkable improvement in pain and disability measures, from week 12 ACS group was superior to steroid groups in VAS, but statistical significance was observed only at week 22 in comparison to the triamcinolone 5 mg group. There was no significant statistical difference between the two steroids groups in 6 months follow-up. The authors concluded that ACS is an effective treatment option for unilateral lumbar radicular compression for pain reduction, and it is potentially superior to steroids [10].

Two studies conducted by Goni and Ravi Kumar *et al.*, assessed the effectiveness of ACS in lumbar and cervical radiculopathy, separately. In a pilot RCT, 40 patients with cervical radicular pain (and VAS = 7 to 10) were randomly allocated to either ACS or methylprednisolone group. Injections were performed fluoroscopy-guided by a senior anesthesiologist, and patients were followed up for 6 months with VAS for pain, neck pain disability scale, neck disability index and Short Form of Health Survey-12 (SF-12). Although all patients showed improvement and no major complications were reported; the ACS group had a gradual and sustained amelioration during the study course, whereas the methylprednisolone group showed some deteriorations over time. This study suggested ACS as an effective and safe option for nonoperative management in cervical radiculopathy patients [42]. The author also studied the efficacy of ACS in the treatment of unilateral single-level lumbar radiculopathy in 20 patients with radiological (x-ray and MRI) signs of disc degeneration and herniation. The injection was performed under fluoroscopy guidance with epidural perineural technique. Patients were evaluated by quadruple VAS, straight leg raising test, revised ODI, and 12-Item Short Form of Health Survey before and after epidural injections at 3 weeks, 3 and 6 months. The results showed significant improvement in all parameters in all follow-up time points after the intervention; the authors concluded this method to be an effective option for lumbar radiculopathy patients [43].

## Conclusion

The high and yet rising prevalence and burden of MSKDs, and also emerging new therapeutic methods merit further investigations about the efficacy and safety of these new treatments including ACS. Joint disorders and specifically OA are the most common diseases subjected to these studies, of which, pieces of evidence of levels I and II are mostly suggesting ACS as safe and effective in pain reduction and function improvement and it is also estimated to have longer-lasting effects.

In conclusion, ACS can be suggested as a safe and effective therapeutic option for joint diseases (especially OA) although the final clinical decision should be made considering individual pathological and socioeconomic characteristics of each case.

There are not enough high-quality human-subject studies evaluating the effectiveness of ACS in soft tissue injuries, although many have been performed on animal models and reported favorable results (such as facilitating the healing process in tendon injuries). There are few human-subject papers and are mostly of inadequate quality regarding small sample size, hence further evaluation is required on this matter.

Another introduced application of ACS is its epidural injection for patients with radiculopathy (nonsurgical cases). Despite the reported significant improvement in symptom relief as the positive therapeutic effects of ACS, regarding the low power and insufficient sample size of these studies, hence, the relatively low-quality evidence provided, the results should be interpreted cautiously and more high-quality investigations, capable of offering strong recommendation, are surely required.

## Future perspective

The newly emerged therapeutic methods of regenerative medicine are increasingly gaining the attention of clinicians and researchers in a wide range of specialties specifically those involved with MSKDs. It is undeniable that the less invasive and also, curative rather than palliative therapeutic options (e.g., injectable blood products in comparison to pain management by oral medications or surgical interventions) are usually preferred by both patients and physicians nowadays.

According to their visibly growing utilization, the literature is also trending toward a further evaluation of these blood products such as PRP and ACS in the last decade. ACS is specifically favorably noticed by some researchers because of its cell-free nature causing fewer allergic reactions, and also its less variable production process. Following its mainly promising results shown in the treatment of diseases like osteoarthritis and tendinopathy, now it is predictable that the clinical applications of ACS will further grow in treating patients with radiculopathy or discopathy, and peripheral nerve entrapments (such as carpal tunnel syndrome). Caudal epidural injections, medial branch block, intradiscal and facet joint injections are estimated to be potential applications for this product in the near future.

In a recent systematic review and meta-analysis study conducted by the author [44], intra-articular injection of ACS was reported to be an effective and safe method for knee osteoarthritis.

Furthermore, with the newly introduced various brands of ACS preparation kits and still growing, the reliability and cost efficacy of this therapeutic method are expected to enhance further.

**Summary points**

- Autologous conditioned serum (ACS) therapy for musculoskeletal disorders has been studied and resulted in varying outcomes.
- The literature has been mainly focused on ACS application in osteoarthritis treatment (mostly involving knee joint), and some high-quality papers reported promising results, introducing ACS as safe and effective for knee osteoarthritis.
- There are a few pieces of research assessing the effectiveness of ACS in soft tissue injuries in human subjects, and despite some positive reports, further research is required on this matter.
- Epidural injection of ACS for patients with radiculopathy also resulted in symptom relief as the positive therapeutic effects of ACS, although regarding the low power and insufficient sample size of these studies, the results should be interpreted cautiously and more high-quality investigations are surely required.
